# Biomarkers in temporomandibular disorder and trigeminal neuralgia: A conceptual framework for understanding chronic pain

**DOI:** 10.1080/24740527.2019.1709163

**Published:** 2020-01-23

**Authors:** Tina L. Doshi, Donald R. Nixdorf, Claudia M. Campbell, Srinivasa N. Raja

**Affiliations:** a Department of Anesthesiology and Critical Care Medicine, Johns Hopkins University, Baltimore, Maryland, USA; b Department of Diagnostic and Biological Sciences, University of Minnesota School of Dentistry, Minneapolis, Minnesota, USA; c Department of Psychiatry and Behavioral Sciences, Johns Hopkins University, Baltimore, Maryland, USA

**Keywords:** facial pain, trigeminal neuralgia, temporomandibular disorder, biomarkers, chronic pain, precision medicine

## Abstract

In this review, we will explore the use of biomarkers in chronic pain, using the examples of two prototypical facial pain conditions: trigeminal neuralgia and temporomandibular disorder. We will discuss the main categories of biomarkers and identify various genetic/genomic, molecular, neuroradiological, and psychophysical biomarkers in both facial pain conditions, using them to compare and contrast features of neuropathic, nonneuropathic, and mixed pain. By using two distinct model facial pain conditions to explore pain biomarkers, we aim to familiarize readers with different types of biomarkers currently being studied in chronic pain and explore how these biomarkers may be used to develop new precision medicine approaches to pain diagnosis, prognosis, and management.

## RÉSUMÉ

Dans cette revue, nous examinerons l'utilisation de biomarqueurs dans la douleur chronique, en utilisant les exemples de deux affections prototypiques de douleur faciale : la névralgie du trijumeau et le trouble temporomandibulaire. Nous discuterons des principales catégories de biomarqueurs et identifierons divers biomarqueurs géétiques/génomiques, moléculaires, neuroradiologiques et psychophysiques pour ces deux pathologies, en les utilisant pour comparer et distinguer les caractéristiques de la douleur neuropathique, non neuropathique et mixte. En utilisant deux modèles distincts de douleur faciale pour explorer les biomarqueurs de la douleur, nous visons à familiariser les lecteurs avec différents types de biomarqueurs actuellement étudiés dans la douleur chronique et à examiner comment ces biomarqueurs peuvent être utilisés pour développer de nouvelles approches de médecine de précision pour le diagnostic, le pronostic et la prise en charge de la douleur.

## The spectrum of facial pain

Facial pain encompasses a broad range of disorders, often presenting significant diagnostic and therapeutic challenges to health care professionals. The estimated lifetime prevalence of facial pain is 26%,^1^ but misdiagnosis and delayed or ineffective treatment of facial pain are exceedingly common. Patients suffering from facial pain may seek consultation from a wide array of specialists, such as dentistry, neurology, otolaryngology, ophthalmology, dermatology, neurosurgery, plastic surgery, oral surgery, pain medicine, sleep medicine, rehabilitation medicine, psychology, psychiatry, physical therapy, and primary care. However, around the world, many of these professionals have little formal training or experience in managing pain, particularly chronic facial pain.^2–6^ Furthermore, a single facial pain condition can present in multiple ways. Features common in one facial pain condition can occasionally present in another, and multiple facial pain conditions may appear in a single patient. Such difficulties in the precise diagnosis and treatment of facial pain can be agonizing, both for the patient and for the clinician, and are illustrative of the challenges of treating chronic pain in general.

**Table 1.** Summary of potential biomarkers discussed in this review

		

TMD = temporomandibular disorder; TN = trigeminal neuralgia; COMT = catechol-O-methyltransferase; 5-HT = serotonin; MRAS = muscle RAS; IL = interleukin; TNF = tumor necrosis factor; CGRP = calcitonin gene-related peptide; SP = substance P; miRNA = microRNA; fMRI = functional magnetic resonance imaging; MRS = magnetic resonance spectroscopy; DTI = diffusion tensor imaging.

The broad spectrum of facial pain disorders is exemplified in two prototypical conditions: temporomandibular disorders (TMD) and trigeminal neuralgia (TN). TMD includes a common group of musculoskeletal and neuromuscular conditions that present as pain or dysfunction related to the temporomandibular joint(s), the muscles of mastication, and/or the associated tissues.^7^ TN is a less common neuropathic pain condition affecting the fifth cranial (trigeminal) nerve. TMD pain encompasses not one pain disorder but several conditions associated with temporomandibular dysfunction and may include difficulties with chewing, speaking, and other orofacial functions.^7,8^ According to the Diagnostic Criteria for Temporomandibular Disorders, history must be positive for “pain in the jaw, temple, in the ear, or in front of the ear,” and “must be modified with jaw movement, function, or parafunction” (p. 9).^8^ The specific diagnosis of pain-related TMD, such as myalgia, myofascial pain, or arthralgia, depends on physical examination of the patient.^8^ Patient-reported location and examiner provocation of the pain are therefore key components in the diagnosis of TMD pain. By contrast, the diagnosis of TN relies heavily on patient description of symptoms. Diagnostic criteria for TN vary slightly across the different guidelines commonly used but generally define TN as severe unilateral paroxysmal pain in the trigeminal distribution that is precipitated by innocuous stimuli.^9–11^ Pain may come and go in an unpredictable fashion, and sensory examination is often normal.^10,11^

Although the underlying pathologies of TMD and TN are distinct, both conditions are clinical diagnoses, and it is not uncommon to misdiagnose one as the other. Both conditions cause facial pain that is often intermittent (but sometimes continuous), usually unilateral (but sometimes bilateral), and frequently precipitated or exacerbated by touch, talking, or eating (but sometimes by nothing at all). TMD pain tends to be described as dull and aching and may radiate to the ears and temporal, periorbital, mandibular, and posterior neck regions.^12^ In contrast, TN is reported as lancinating, electric, and shooting in the distribution of the trigeminal nerve.^13^ However, symptoms in both conditions can be variable and may change over time.^14^ It is increasingly recognized that a high proportion of patients with TMD and headaches suffer simultaneously from multiple other pain conditions, and the term “chronic overlapping pain conditions” has been introduced to suggest possible shared etiology and disease mechanisms.^15^ By contrast, TN is limited to the trigeminal nerve, and though other pain conditions may also occur concurrently, it is not as common as in TMD. In addition, the prognosis of TMD pain is fairly good, with only 5% to 10% of those with symptoms requiring treatment and a spontaneous resolution rate of up to 40%,^12^ whereas TN can be unpredictable, with periods of remission and recurrence lasting weeks to years over the course of a lifetime.^13^

The subjective nature of pain, combined with the evolving, overlapping, and often complex features of both types of pain, highlights the need for objective markers of chronic pain. Such biological markers, or biomarkers, can aid in the correct diagnosis, treatment selection, and prognosis of chronic pain disorders. In this review, we will familiarize the reader with potential biomarkers in TMD and TN (Table 1). It is not intended to be comprehensive list of all biomarkers in facial pain. (For a more in-depth exploration of this topic, we recommend an excellent textbook by Goulet and Velly.^16^) Through the two distinct prototypical facial pain disorders of TMD and TN, we are provided with a useful context in which to understand the promise and pitfalls of biomarkers in chronic pain.

## The role of biomarkers in chronic pain

The hunt for biomarkers in chronic pain has intensified in recent years, as interest has grown in personalized/precision medicine techniques, and the global opioid crisis has underscored the need to accelerate the pace of pain research. In late 2018, the U.S. National Institutes of Health and National Institute of Neurological Disorders and Stroke convened a workshop of international experts in pain research to recommend best practices in pain biomarker discovery and validation, which “would help to define pathophysiologic subsets of pain, evaluate target engagement of new drugs, and predict analgesic efficacy of new drugs” (para. 3).^17^ Their published recommendations are forthcoming, but despite the group’s extensive discussions and enthusiasm for promising avenues of research, there remain no objective, measurable biomarkers for the detection and quantification of pain. State of the art in pain management, particularly in facial pain management, continues to rely on patient self-report, clinical diagnosis, and clinical decision making.

**Figure 1.**
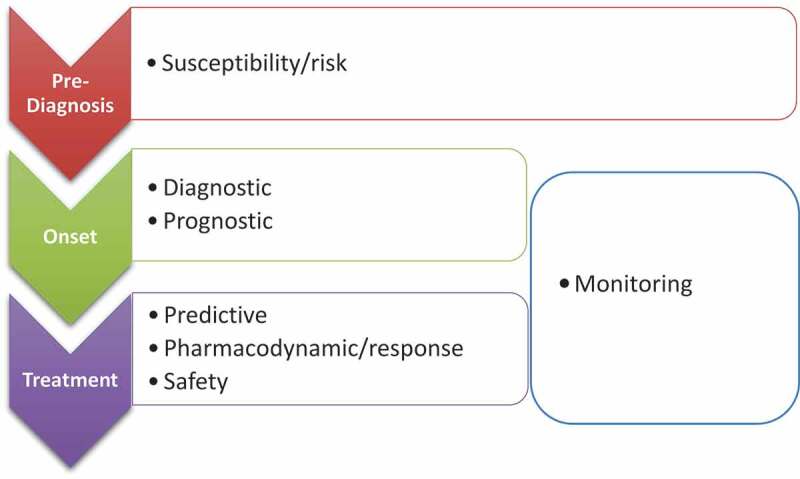
Types of biomarkers along the disease trajectory

The term “biomarker” is often misinterpreted as any variable that can be quantified and applied to characterize a disease state. However, demographic data, patient-reported outcome measures, and environmental exposures are not biomarkers, because they do not accurately and reliably correspond to an individual’s health. In 2001, the World Health Organization defined a biomarker as “any substance, structure or process that can be measured in the body or its products, and influence or predict the incidence or outcome of disease” (p. 1).^18^ The BEST (Biomarkers, EndpointS, and other Tools) resource developed as a joint effort between the U.S. Food and Drug Administration (FDA) and the U.S. National Institutes of Health defines a biomarker as “a defined characteristic that is measured as an indicator of normal biological processes, pathogenic processes, or responses to an exposure or intervention, including therapeutic interventions … not an assessment of how an individual feels, functions, or survives” (p. 41).^19^ BEST also classifies biomarkers into several categories, and they may be detected anywhere along the trajectory of the disease or its management (Figure 1). Susceptibility/risk biomarkers portend the onset of disease, diagnostic biomarkers confirm its presence, prognostic biomarkers forecast the course of the disease, predictive biomarkers relate to the potential response of the disease to intervention or exposure, pharmacodynamic/response and safety biomarkers characterize those interventions or exposures, and monitoring biomarkers track trends in these other biomarkers over time.^19^

**Figure 2.**
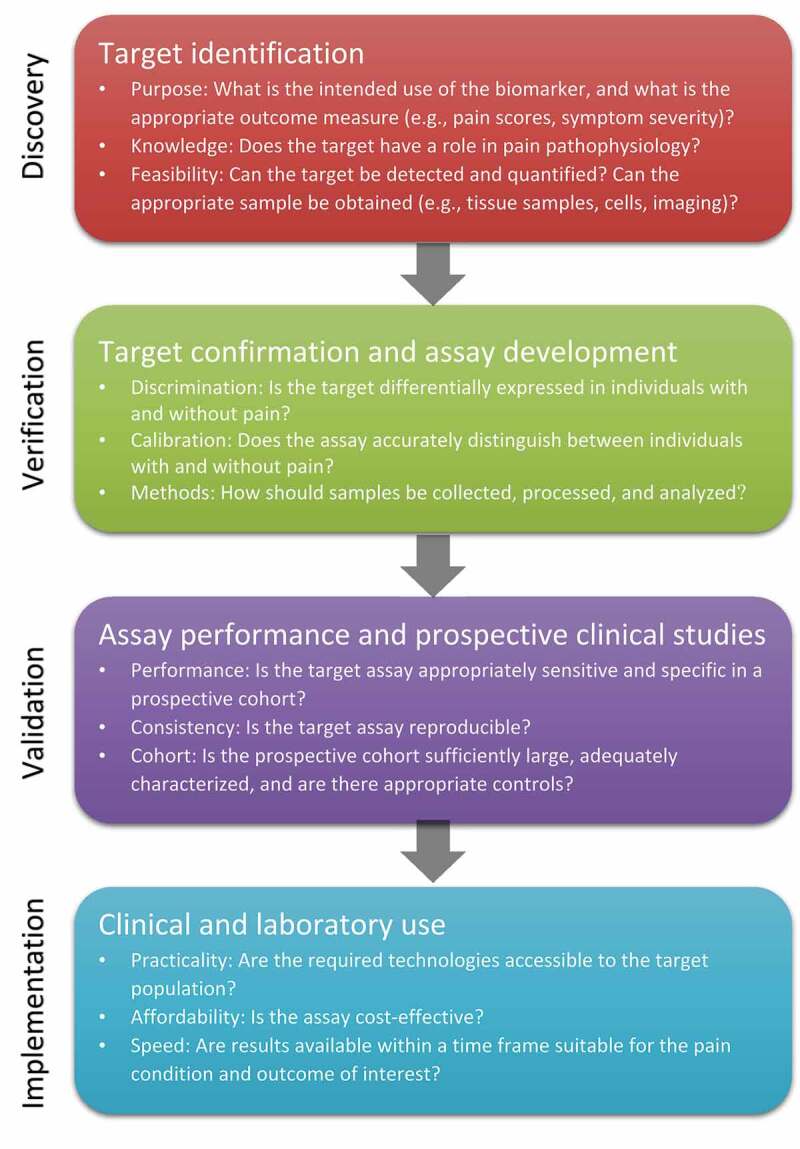
Framework for assessment of potential biomarkers in chronic pain

A general framework for the evaluation of potential biomarkers along each step of the development pathway, placed in the context of chronic pain, is provided in Figure 2. An ideal biomarker must clearly distinguish between individuals with or without the condition of interest (good discrimination), and this distinction must be accurate (good calibration). The defined threshold values that separate positive from negative outcomes must have an appropriate balance of sensitivity and specificity depending on the biomarker’s intended application (e.g., screening, diagnosis, prediction, or prognosis). The ideal biomarker must also be easily and affordably detected and measured, with consistent, reproducible results across the biological variability of the condition of interest. Although there are currently no ideal biomarkers for facial pain (or, indeed, any type of pain), recent research has focused on potential biomarker candidates, including genetic/genomic, molecular, neuroradiological, and psychophysical biomarkers.

Although candidate biomarkers may initially be identified from animal studies, small observational cohorts, or case–control studies, biomarker validation typically requires large samples of patients and clinical data sets. Prospective cohort studies are valuable because they reduce the risk of recall bias compared to retrospective cohorts and can be designed to track specific exposures and outcomes of interest, and large cohorts increase the likelihood of finding a significant association between a putative biomarker and a relatively rare outcome (e.g., onset of a chronic pain condition).^20^ The Orofacial Pain: Prospective Evaluation and Risk Assessment (OPPERA) study was the first and largest prospective study designed to examine and identify biopsychosocial, environmental, and genetic factors contributing to the development of TMD.^21^ Subsets of other large cohorts assembled around the world have also been used to study TMD, including the Study of Health in Pomerania in Germany,^22^ the Hispanic Community Health Study/Study of Latinos in the United States,^23^ and the United Kingdom Biobank.^24^ Such TMD cohorts have allowed researchers to pool resources internationally and explore a variety of potential biomarkers, such as genetic and psychophysical associations in TMD.^25,26^ For TN, no similar cohorts exist, and the relatively low incidence of TN (about 4 in 100,000)^27^ makes assembling a TN cohort much more challenging. Many biomarker studies in TN have relied on miniscule sample sizes and small retrospective cohorts. However, a recent effort from the Facial Pain Research Foundation (https://www.facingfacialpain.org/index.php) is underway to create a database of TN patients, and biomarker studies may develop from this cohort in the future.

## Genetic biomarkers

A person’s genetic code can serve as a pain biomarker in a variety of ways. Single nucleotide polymorphisms (SNPs) in a gene can have a profound impact on its function and its role in the development of chronic pain. Genes may interact with each other or with the environment, altering gene expression to make an individual more or less likely to develop a chronic pain condition. Epigenetic regulation can also modulate gene expression, altering gene function without disturbing the underlying genetic code. Each step in the sequence from genetic code to gene expression is a potential source for biomarkers.

One of the most widely studied genetic biomarkers in facial pain is catechol-O-methyltransferase (COMT) in TMD. COMT encodes a ubiquitous enzyme responsible for the metabolism of catecholamines, which include the neurotransmitters dopamine, epinephrine, and norepinephrine. Several SNPs in the *COMT* gene have been identified in individuals with TMD, with varying combinations of these SNPs comprising haplotypes that fall into one of three categories: low pain sensitivity (LPS), average pain sensitivity, and high pain sensitivity.^28^ Individuals with the LPS haplotype have higher levels of *COMT* enzymatic activity, which is in turn associated with a 2.3 times decreased probability of developing TMD. In a prospective cohort study, Slade et al. found that women with average pain sensitivity or high pain sensitivity haplotypes had a 2.7-fold increased relative risk of developing TMD following orthodontic treatment compared to those with “pain-resistant” LPS haplotypes.^29^ Although recent studies have since found little to no evidence that orthodontic treatment is a risk factor for TMD,^30,31^ the Slade et al. study did demonstrate that genotype may be associated with development of TMD following orthodontic treatment. Subsequent studies have provided further evidence that certain *COMT* genotypes may be significant risk or protective factors in the development of TMD.^32–36^ However, research has also indicated that no single gene is responsible for TMD. A systematic review of family and genetic association studies in TMD concluded that TMD heritability is multifaceted, with the most evidence for contributions from genes encoding proteins involved in the serotonergic and catecholaminergic systems.^37^ The OPPERA study identified associations between TMD and SNPs in COMT, the serotonin receptor HTR2A, and the glucocorticoid receptor NR3C1 (the binding site for cortisol), among others, but, notably, the researchers needed to combine the OPPERA data with those from a separate cohort including 182 TMD cases and 170 healthy controls in order for any of the identified associations to reach statistical significance.^38^ More recently, the OPPERA researchers pooled two separate cohorts of TMD patients and controls and found a 2.9 times increased odds of TMD in men (but not women) possessing an SNP in chromosome 3 that decreases expression of the muscle RAS oncogene homolog gene, which is involved in cell growth and differentiation processes.^25^ These findings were nominally replicated in the researchers’ meta-analysis of seven other TMD cohorts but were not statistically significant.^25^

Genome-wide association studies suggest that genetic and epigenetic factors may be implicated in chronic widespread pain conditions, such as fibromyalgia, that often overlap with TMD. Potential candidate genes identified include *SLC64A4, TRPV2, MYT1L*, and *NRXN3*.^39^ Environmental factors and early life experiences may also modulate genome function through epigenetic mechanisms. Epigenetic changes in women with fibromyalgia have been identified using genome-wide methylation pattern analysis.^40^ It is also important to note that comorbid conditions may confound or modify the relationship between candidate biomarkers and orofacial pain; biomarker identification therefore requires careful patient selection and/or appropriately adjusted statistical analyses.^41^ Further studies are also needed to examine whether similar changes are observed in patients with TMD without widespread pain conditions.

The challenges of genetic biomarker validation are compounded many times over for a rare disease like TN, but a unique subtype of TN suggests that there may be a stronger link between genes and TN than between genes and TMD. Although most TN cases occur sporadically, familial trigeminal neuralgia is a well-documented phenomenon. No single genetic locus has been identified in all cases of familial TN, and analyses of various familial TN lineages have reported both autosomal recessive^42^ and autosomal dominant^43^ inheritance patterns. These observations strongly suggest that TN may be associated with genetic factors in some patients.^44^ One study of 244 TN patients found that an SNP in the promoter region of the serotonin transporter (5-HTT) resulted in decreased levels of 5-HTT expression and corresponded with increased risk of having TN, higher pain severity, and poorer response to carbamazepine.^45^ In another study, researchers identified a gain-of-function mutation in the sodium channel Na_v_1.6 in one individual with TN.^46^

As in TMD, there is some evidence relating the genetics of the descending pain modulatory pathways of the central nervous system to the development of TN. However, the handful of animal studies exploring trigeminal pain in knockout mice suggest that TN may be associated with mutations in genes encoding voltage-gated ion channels and regulators of cellular signaling and neuroinflammation, which is hardly surprising in a neuropathic pain condition.^44^ Unfortunately, none of these animal studies have translated to human genetic associations. A 2009 Brazilian study reported findings that Na_v_1.7 was downregulated and Na_v_1.3 was upregulated in patients with TN compared to healthy controls.^47^ This study was considerably underpowered, with only 13 patients in each group, and even though a genetic polymorphism mechanism was proposed, no specific SNPs were identified. A more recent study of 48 TN patients and 48 controls, also in Brazil, did not find any association between polymorphisms in Na_v_1.7 and the nerve growth factor receptor TrkA.^48^ As more research emerges in selective sodium channel blockers for the treatment of TN,^49^ genetic variants of sodium channels may also be identified that could serve as predictive biomarkers for this potential TN therapy.

Perhaps the most significant genetic biomarkers in TN are related not to its development, diagnosis, or prognosis but to the safety of its treatment. The HLA-B*1502 allele, most commonly found in individuals of East Asian descent, predicts up to a tenfold increased risk of severe or fatal skin reactions (e.g., Stevens-Johnson Syndrome, toxic epidermal necrolysis) following exposure to the anticonvulsant carbamazepine.^50^ Similarly, the HLA-A*3101 allele, found in most populations worldwide, is associated with Stevens-Johnson Syndrome/toxic epidermal necrolysis and other serious carbamazepine-induced drug reactions.^51^ Carbamazepine is considered first-line therapy for TN and is the only medication approved by the FDA for the treatment of trigeminal neuralgia. However, drug safety organizations around the world, including the FDA, the Royal Dutch Association for the Advancement of Pharmacy, the Clinical Pharmacogenetics Implementation Consortium, and the Canadian Pharmacogenomics Network for Drug Safety, have all published prescribing and dosing guidelines for carbamazepine based on genotype.^52^ In general, genetic testing prior to initiation of carbamazepine is recommended for all patients from populations in which the frequency of these genotypes is common or unknown or if a previously untested patient develops a serious drug reaction after starting on the drug. For patients who test positive for a high-risk genotype, alternative medications are strongly recommended.

## Molecular biomarkers

Molecular biomarkers are attractive research targets. Depending on a study’s particular aims and methodology, a single assay from a simple cheek swab, blood test, or biopsy could provide diagnostic, prognostic, predictive, or monitoring information. In addition, unlike genetic, psychophysical, or radiological biomarkers, which are typically observed rather than manipulated, many molecular biomarkers are enticing pharmacologic targets. However, the complex, heterogeneous, and often nebulous mechanisms underlying chronic pain conditions make it much more difficult to find good molecular biomarkers for pain, whereas diseases associated with more concrete entities (e.g., single-gene mutations, viruses, nutritional deficiencies) offer more obvious targets as potential biomarkers. Nevertheless, understanding the pathophysiology of disease in TN and TMD is a logical starting point in the search for molecular biomarkers in facial pain.

TN pain arises from dysfunction of the trigeminal nerve. Vascular compression of the trigeminal nerve is present in approximately 95% of patients with TN, and most evidence suggests that compression of the nerve root leads to focal demyelination and/or hyperexcitability of the nerve, causing the distinctive features of TN.^13^ Most other cases of TN without vascular compression occur in neurodegenerative lesions, such as multiple sclerosis plaques or lacunar infarcts of the trigeminal nerve root, which are also associated with demyelination and hyperexcitability.^13^ Central mechanisms may also play a role: neurophysiologic studies have found impaired inhibition of central nociceptive pathways in patients with TN with concomitant chronic facial pain, a substantial population of patients with TN who are particularly refractory to treatment.^53^

TMD does not have a single anatomic origin; broadly speaking, it may arise from degeneration of the temporomandibular joint (TMJ), painful TMJ disc displacement, and pain within the muscles of mastication. TMJ degeneration may occur through various pathologies, such as osteoarthritis, degenerative joint disease, or autoimmune arthritis, as well as through exacerbation by mechanical stressors.^54^ Mechanical stimulation of nociceptors results in increased levels of neuropeptides and inflammatory mediators and local hypoxia; these changes can lead to pain and dysfunction, potentially exacerbating degeneration and mechanical stress on the joint^54^ but also for the muscles of mastication.^55,56^ Thus, TMD encompasses joint-related pain as well as associated myalgia, myofascial pain, tendonitis, spasm, and myositis.^8^ Furthermore, as with many other chronic pain conditions, prolonged peripheral stimulation of nociceptive pathways can lead to central sensitization of temporomandibular pain; consequently, patients with TMD fall along a continuum from peripherally generated to centralized pain.^57^ In addition, as discussed earlier, a substantial proportion of individuals with TMD present with other chronic overlapping pain conditions, such as irritable bowel syndrome, migraine headaches, fibromyalgia, and pelvic pain. A common characteristic in these patients is sensory hypersensitivity and pain amplification, suggesting a central sensitization mechanism.^15^

The simplistic perspective that TMD is a problem of inflammation and sensitization, whereas TN is a problem of nerve dysfunction, would suggest very different areas of biomarker research for the two conditions. However, chronic pain is never that simple, and some evidence suggests that inflammation could contribute to TN pain,^58,59^ and nerve dysfunction may play a role in TMD.^60,61^ Moreover, the same nerves can be involved in both conditions. Sensory innervation of the temporomandibular joint is supplied by the V_3_ branch of the trigeminal nerve, so in cases of TMD involving facial or joint pain, the trigeminal nerve is necessarily involved in pain transmission. The branches of the trigeminal also supply motor innervation to the muscles of mastication (masseter, temporalis, medial, and lateral pterygoids and anterior digastric); therefore, dysfunction of the trigeminal nerve may also lead to dysfunction of musculoskeletal structures involved in TMD. Identifying areas of overlap and dissimilarity in biomarkers for the two conditions has important implications for understanding which molecules may better serve as screening or diagnostic biomarkers and which are better suited as predictive biomarkers.

### Cytokines and other inflammatory mediators

Although both TMD and TN may cause pain extending beyond the pathological anatomic structure, they are also both characterized by pain that is localized to the craniofacial region. As such, researchers intuitively seek biomarkers that are also localized to the specific area of interest. As a superficial structure, the temporomandibular joint is significantly more accessible than the trigeminal nerve. The prospect of obtaining salivary, synovial, or even muscle biopsy samples for TMD biomarker research is more appealing (and less daunting) than the analogous collection of cerebrospinal fluid (CSF) or nerve biopsy from the trigeminal nerve. Consequently, molecular biomarker research in TMD covers a wide range of bodily tissues, from blood to biopsy, whereas relatively few TN studies have analyzed more than blood and, occasionally, CSF.

Research on inflammatory mediators in facial pain provides an excellent illustration of this challenge. The strongest evidence of differences in inflammatory profiles in patients with TMD pain compared to controls has been in synovial fluid rather than in plasma. Increased levels of the pro-inflammatory cytokine tumor necrosis factor (TNF) have been found in the synovial fluid of patients with TMD,^62–65^ and higher levels of synovial TNF-α are associated with increased TMD pain.^66^ Synovial TNF-α levels have also been found to be predictive of treatment response to intra-articular glucocorticoid injection^67^ and temporomandibular joint surgery,^68^ with degree of pain relief corresponding to concomitant decreases in TNF-α levels after the procedure. Other cytokines studied in TMD include the pro-inflammatory cytokine interleukin-1β (IL-1β) and the mixed-effect cytokine IL-6. Like TNF-α, IL-1β is increased in the synovial fluid of patients with TMD,^65,69^ and increased synovial fluid IL-1β is associated with increased TMD pain.^63,70^ Similarly, IL-6 is found more frequently in the synovial joints of patients with TMD compared to healthy controls, and higher levels are correlated with increased pain and TMD symptoms.^65,69,71–73^ Beyond synovial fluid, intramuscular cytokines and salivary biomarkers have also been studied in TMD. For example, elevated levels of IL-6, IL-7, IL-8, and IL-13 have been found in the masseter muscles of patients with TMD myalgia,^56^ and salivary levels of oxidative stress biomarkers 8-hydroxydeoxyguanosine and malondialdehyde and total antioxidant status have been found to be significantly different between patients with TMD and controls.^74^

A growing body of evidence indicates that inflammation may play a role in the development of neuropathic pain,^75^ but there are few studies of cytokines in TN. A recent study collected venous blood from patients with TN and hemifacial spasm (a similar pathological condition affecting the facial nerve) and found increased concentrations of IL-1β, IL-6, IL-8, and TNF-α compared to healthy volunteers.^59^ However, all samples were collected during microvascular decompression (MVD) surgery, and it is unclear how the environmental stress of the perioperative setting may have affected cytokine levels. Cytokine analysis of the CSF surrounding the trigeminal nerve may be less influenced by environmental factors, but only one study reporting on a technique to measure cytokines in the CSF of patients with TN has been published.^76^ Although cytokines were detected, mediator levels varied depending on where the sample was collected along the trigeminal nerve root, and no comparisons could be made to a control population.

The two examples of cytokine profiles in TMD and TN illustrate some of the difficulties in using cytokines as biomarkers. First, it is worth noting that cytokine studies typically evaluate levels of multiple cytokines, but in many of the studies referenced above only one or two cytokines were found to be significantly different between patients and controls. Alstergren et al. have recently proposed clinical diagnostic criteria for temporomandibular joint arthritis by measuring synovial fluid levels of seven different inflammatory mediators; a concentration above normal for any one of these inflammatory mediators was considered diagnostic of arthritis.^77^ However, it is unclear how the specific mediators chosen are any more or less reflective of TMD pathology than any of the dozens of other mediators that have been studied, and the high cost of multiplex cytokine assays may limit the use of this approach. Second, cytokine levels are known to vary widely within individuals. Basi et al. assayed venous blood, biopsied masseter muscle, and temporomandibular joint synovial fluid from patients with TMD and healthy controls for levels of the inflammatory mediators bradykinin, F2-isoprostane, leukotriene B4, nerve growth factor, prostaglandin E2, and substance P.^78^ Although muscle levels of F2-isoprostane were negatively correlated with muscle pain intensity and pressure pain threshold, no other biomarkers were correlated with pain intensity. Furthermore, only plasma bradykinin was correlated with synovial bradykinin levels, and there were no significant correlations among the tissue types for any of the other mediators. Thus, it appears that inflammatory mediator levels are highly dependent on sample location, particularly in localized disease states such as TMD. In addition, levels of cytokines and inflammatory mediators may fluctuate according to time of day, fasting status, physical activity, and stress.^79^ Taken together, these findings suggest that although cytokines and other inflammatory mediators may provide insights into the mechanisms of pain in TMD and TN, and perhaps a broad sense of inflammation in patients with TMD and TN, they may be too variable and unpredictable to serve as practical biomarkers in facial pain.

### Neuronal signaling molecules: Neurotransmitters and neuropeptides

Whether pain is neuropathic or nociceptive, all pain signals must be conducted through the nervous system. Neuronal signaling molecules therefore play a key role in pain processing and have been explored as potential molecular biomarkers in a variety of chronic pain conditions. In chronic facial pain, most of this research has focused on the monoamine neurotransmitters and neuropeptides. The monoamine neurotransmitters include serotonin (5-HT), dopamine, and norepinephrine, whose roles in the descending inhibition and affective components of chronic pain have been studied extensively yet are not completely understood. Neuropeptides, which are often co-secreted with neurotransmitters, are released by neuronal cells to facilitate intercellular communication. These small molecules, such as calcitonin gene-related peptide (CGRP), substance P (SP), and nerve growth factor, are vital to the initiation and amplification of a variety of inflammatory, nociceptive, and vasoactive processes, including neurogenic inflammation. The best example of a validated biomarker in craniofacial pain is CGRP in migraine, the levels of which are elevated in blood and saliva during migraine attacks.^80^ The prospective utility of these signaling molecules as biomarkers is further enhanced by the availability of targeted pharmacological therapies that are already in clinical use, including 5-HT modulators, norepinephrine reuptake inhibitors, α_2_ agonists, and CGRP receptor antagonists. CGRP receptor antagonists have been recently approved for the prevention of migraine episodes and are an excellent illustration of how biomarker research can have a profound impact in our understanding of pain and its treatment.

In TMD, elevated synovial 5-HT has been associated with increased pain,^81^ and both local and systemic levels of 5-HT predict response to intra-articular glucocorticoid injection.^82^ A small study found that the masseter muscles of women with myofascial TMD had more nerve fibers expressing 5-HT_3A_ receptors compared to healthy controls and that these fibers more frequently exhibited increased co-expression of 5-HT_3A_ receptors with Na_v_1.8 channels, a marker of small, thinly myelinated nociceptive fibers.^83^ A more recent study found no difference in plasma levels of 5-HT between patients with myofascial TMD and healthy controls but found that plasma dopamine levels were significantly increased in patients with TMD.^84^ These findings suggest a complex relationship between the peripheral and central actions of these neurotransmitters in TMD pain.

Neuropeptides may also contribute to TMD pathophysiology. Both CGRP^85^ and SP^86,87^ have been found to be increased in synovial samples from patients with TMD pain, but only CGRP levels are positively correlated with pain. These observations suggest that SP levels may reflect joint injury or pathology, whereas CGRP levels may be more reflective of joint pain. However, it not clear whether these associations reflect increased expression in response to mechanical joint injury or whether these neuropeptides, which are both potent vasodilators, are secreted in response to local tissue hypoxia that may occur in TMD.^54,88^

As previously noted, due to the challenges of studying the local milieu of the trigeminal nerve, biomarker studies in TN are relatively rare. The few available studies create a slightly different picture of our understanding of TN pain compared to TMD. Whereas in TMD 5-HT was found to be elevated and associated with increased pain, rodent models of TN have found that agonism of 5-HT_1A_ and 5-HT_2C_ receptors attenuates pain behaviors.^89,90^ Serotonin (5-HT) has a complex role in pain. Outside of the central nervous system, 5-HT acts as an inflammatory mediator and sensitizes afferent nerve fibers to induce hyperalgesia.^91^ Inside the central nervous system, it can have analgesic or hyperalgesic effects in the brainstem and spinal cord, inhibit neurotransmitter release in the trigeminal system, or modulate descending pain inhibition pathways.^91^ Thus, the particular effects of serotonin will depend on where it is located in the body, the relative concentrations, and available receptor subtypes, meaning that serotonin modulators may have very different effects in TMD versus TN, depending on route of administration, dosing, and receptor selectivity.

Only a handful of other neuronal signaling molecules have been studied in patients with TN. A study of CSF samples from 16 patients with TN found that the concentrations of norepinephrine and its metabolite vanillylmandelic acid, the dopamine metabolite homovanillic acid, the serotonin metabolite 5-hydroxyindoleacetic acid, and somatostatin were all significantly decreased compared to controls, whereas SP was increased.^92^ The authors suggested that elevated SP might indicate neurogenic inflammation, whereas changes in the monoaminergic systems might reflect central dysfunction in TN. Consistent with this hypothesis, a subsequent study comparing the CSF of 20 TN patients with that of 20 controls with nervous system or gynecological disease found that the neuropeptides CGRP, SP, and vasoactive intestinal peptide were significantly elevated in patients versus controls, whereas β-endorphin was significantly decreased.^93^

### “Omics” profiling

Recently, exploration of the human genome, epigenome, transcriptome, proteome, and metabolome has become possible with the availability of reliable high-throughput technologies, sparking increased interest in so-called omics biomarkers. Researchers can now extract prodigious quantities of information from a single patient, or even a single cell, to develop a comprehensive, personalized biomarker profile. This approach allows many potential biomarkers to be studied at the same time from very small sample quantities. Data from these RNAs, proteins, or metabolites provide information about the function and functionality of entire pathways, giving investigators a perspective on disease that is both broad and detailed. However, the information obtained is only as valid as the source of the information; poor patient selection, poor sample selection, and poor sample collection may all yield misleading results. Researchers must also guard against the trap of equating statistical significance with clinical significance. Analyzing the sheer volume of data produced from these assays requires advanced statistical and computational skills, but even an excellent statistical analysis can fail to produce useful biomarkers. Consequently, the identification of valid, practical biomarkers requires an approach that balances statistical rigor with expert knowledge about the scientific underpinnings of disease.^94^

Although omics profiling is now more readily available than ever before, assays are still quite expensive, time and labor intensive, and computationally complex. As a result, there are few published studies evaluating these biomarker profiles in TMD or TN. MicroRNA (miRNA) profiling of synovial fibroblasts from patients with TMD found decreased expression of the miRNA221-3p.^95^ miRNAs are small, noncoding RNA molecules that act as regulators of gene expression. The researchers found that miRNA221-3p inhibits transcription of Ets-1, which is itself a transcription factor for matrix metalloproteinases (MMP). MMPs include a large family of enzymes responsible for tissue degradation and remodeling, particularly in joint cartilage. IL-1β reduces miRNA221-3p, upregulating Ets-1 and its downstream MMP products. This example provides a good illustration of how omics profiling may lead to insights on the mechanisms of joint degeneration in TMD. Recently, a rapid biomarker-based method has been reported, using vibrational spectroscopy for metabolomic analysis of blood smears, which could serve as a metabolic fingerprint to differentiate patients with fibromyalgia from those with other rheumatologic disorders.^96^ Whether such tools can be used for the diagnosis of craniofacial pain states remains to be determined.

In TN, a preliminary study examined the plasma proteome of patients before and after MVD surgery, a neurosurgical procedure used to treat TN, and compared patients to healthy volunteers.^97^ Patients had significantly altered levels of several proteins, including transthyretin, retinol binding protein, and alpha-1-acid glycoprotein 2, proteins that may play a role in oxidative stress and peripheral nerve regeneration. In addition, the investigators found alterations in plasma levels of CGRP, nitric oxide, glycine, and vitamin D before versus after surgery, suggesting that these molecules may play a role in pain sensitization. To our knowledge, no other published studies have examined omics biomarkers in TN. However, as these technologies become more accessible over time, more studies in TMD and TN will undoubtedly emerge.

## Neuroradiological biomarkers

Neuroradiological imaging is another promising area of pain biomarker research. Advances in imaging techniques over the past several decades have allowed better quantification of the structural, chemical, cognitive, and psychological changes that occur in chronic pain. Neuroimaging in chronic pain has many potential benefits, including noninvasive characterization of brain structure and function (e.g., magnetic resonance imaging [MRI]); assessment of changes that occur in response to various stimuli and tasks and cognitive and behavioral states (e.g., functional MRI [fMRI]); evaluation of pharmacologic function (e.g., positron emission tomography); and evaluation of neurotransmitter and metabolite concentrations (e.g., magnetic resonance spectroscopy [MRS]).^98^ However, these techniques require a substantial investment of equipment, expense, participant burden, and image interpretation and analysis, and studies can be limited by artifacts, low resolution, risks of the imaging techniques (e.g., use of radiotracers in positron emission tomography, contraindications to MRI), and challenges in making causal inferences based on observed group differences.^98^ Nevertheless, neuroimaging remains a powerful potential tool in pain research, with new discoveries in forward translating pain mechanisms into potential diagnostics or treatments or reverse translating clinical observations to refine preclinical models of chronic pain.^99^

Numerous studies have investigated neuroimaging in TMD. Structural MRI studies have found evidence of white matter abnormalities in the trigeminal nerve and corpus callosum of patients with TMD compared to controls, suggesting that increased nociceptive activity in TMD may cause microstructural changes in the trigeminal nerve and be associated with changes in sensory, motor, cognitive, and pain pathways.^60^ Similarly, patients with TMD have been found to have abnormalities in gray matter in brain areas associated with pain, modulation, and sensorimotor functioning, and these changes are correlated to pain duration, intensity, and unpleasantness.^100^

In a comprehensive literature review of neuroimaging in several “central sensitivity” syndromes, including TMD, Wallitt et al. concluded that there were inconsistent and conflicting data regarding basal neuronal activity patterns (*n* = 3 studies), gray matter volume (*n* = 3), and white matter volume (*n* = 2) in structural MRI studies of patients with TMD.^101^ All TMD studies included in the review examined fewer than 20 (and in some cases, fewer than 10) patients with TMD, and it may be that the observed inconsistencies derive from small sample sizes and the clinical heterogeneity of TMD. Functional MRI studies have provided some evidence demonstrating that patients with TMD have changes in cortical processing that manifest as increased sensitivity to nonpainful tactile stimuli.^102,103^ In addition, the only molecular measurement study published at the time of the review demonstrated increased glutamine in the right posterior insula and increased N-acetylaspartate and choline in the left posterior insula in MRS of patients with TMD.^104^ A more recent MRS study found increased total creatine in the posterior insula of patients with TMD and, furthermore, that increased choline and glutamate concentrations in the posterior insular cortex were correlated with clinical characteristics of TMD pain, including generalized pain.^105^

Because MRI is a standard diagnostic tool in TN and is critical to evaluation for surgical treatments of TN, almost all patients with TN undergo neuroimaging. However, research MRIs often use specialized protocols and postprocessing techniques not typically used in clinical practice. In TN research, high-resolution anatomical imaging (e.g., variations of T1- and T2-weighted images) assesses anatomical characteristics and patterns of neurovascular contact, brain gray matter volume, and cortical thickness, and diffusion imaging (e.g., diffusion-weighted images, diffusion tensor images [DTI]) assesses brain white matter and trigeminal nerve microstructure.^106^ These advances in neuroimaging have already had significant practical implications in TN. Until recently, a central debate in our understanding of TN pathophysiology has been the degree to which neurovascular contact explains TN symptoms. Although contact of the trigeminal nerve by an overlying blood vessel is one of the most common explanations for pain in TN, it has also been observed that neurovascular contact is not always present in patients diagnosed with TN.^106^ Furthermore, contact of vessels with the trigeminal nerve can often be seen on routine biopsy and conventional MRI of asymptomatic individuals.^106^ However, advanced imaging techniques have provided better resolution of the trigeminal nerve, demonstrating that although neurovascular contact may occur in asymptomatic trigeminal nerves, symptomatic trigeminal nerves are frequently dislocated or distorted by compressive vascular structures; furthermore, those changes correlate with TN symptom severity.^107–109^ Neuroradiological biomarkers have also been used to predict treatment response to TN treatments. DTI uses the restricted diffusion of water in tissues to provide detailed information about trigeminal nerve microstructures. Pretreatment DTI metrics have been correlated with treatment response of patients following TN surgery, such as MVD,^110^ stereotactic radiosurgery,^110^ and radiofrequency lesioning.^111,112^

The utility of diffusion-weighted imaging and DTI goes beyond TN and they have also been used to study other chronic orofacial pain conditions, including TMD, yielding a better understanding about the neural mechanisms underlying trigeminally mediated pain.^113^ In a study using high-resolution MRI and DTI, Wilcox et al. found that patients with TN had a significant decrease in nerve volume compared to controls, patients with neuropathy had a significant increase in nerve volume, patients with TMD displayed no difference in volume, and none of the patient groups demonstrated significant changes in DTI values.^114^ A review of brain signatures in chronic orofacial pain examined neuroimaging studies in TMD and trigeminal neuropathic pain (TNP), which includes TN, posttraumatic trigeminal neuropathy, and postherpetic neuropathy.^115^ TNP disorders have different underlying etiologies, but all involve dysfunction of the trigeminal nerve and have very similar clinical features. Summarizing the available studies, the author concluded that patients with TMD and TNP demonstrated consistent structural and functional changes in the thalamus and the primary somatosensory cortex, as well as the prefrontal cortex and the basal ganglia, indicating the importance of the thalamocortical pathway, cognitive modulation, and reward processing in chronic orofacial pain. However, it also appeared that patients with TNP had greater alterations to the thalamocortical pathway and, furthermore, that the two conditions displayed different patterns of thalamic and insular connectivity.^115^ Subsequent studies have supported these differences. Youssef et al. examined cerebral blood flow using fMRI in patients with TMD (nonneuropathic pain) and TNP (neuropathic pain).^116^ Neuropathic pain was associated with decreased cerebral blood flow in the thalamus, primary somatosensory, and cerebellar cortices. Nonneuropathic pain was associated with significant increases in cerebral blood flow to the anterior cingulate cortex, the dorsolateral prefrontal cortex, and the precuneus—regions generally associated with higher-order cognition and emotion—as well as motor-related regions and the spinal trigeminal nucleus.^116^ Although this report suggests that neuroradiological biomarkers may help distinguish between neuropathic and nonneuropathic pain, these observations should be interpreted with caution. Differences in neuroradiological biomarkers may demonstrate different changes associated with each condition, but they are not necessarily reflective of differences in underlying mechanisms of pain. As we will discuss next, quantitative sensory studies of patients with TMD suggest that sensory amplification occurs in many patients, indicating central sensitization (a hallmark of neuropathic pain) as a mechanism of pain in TMD.

## Psychophysical biomarkers

Quantitative sensory testing (QST) is a noninvasive psychophysical method using calibrated, objective stimuli (e.g., heat, touch, pressure, vibration) to elicit subjective patient responses (e.g., detection of sensation or pain). Depending on the stimulus used, QST can evaluate loss and gain of function along large (Aβ) or small (Aδ, C) fiber pathways from various peripheral body sites to the somatosensory cortex. Although frequently used in pain research, QST is rarely used in clinical practice, in part due to equipment costs and time involved in conducting the tests. Performing QST also requires specialized training, and wide variations between subjects, QST protocols, and examiners make it difficult to interpret results. In recent years, standardized QST protocols have been developed, along with age-, gender-, and site-associated reference values in healthy volunteers.^117–119^ The International Association for the Study of Pain Neuropathic Pain Special Interest Group released a consensus statement in 2013 recommending QST for screening for small and large fiber neuropathies, monitoring somatosensory deficits, and monitoring of evoked pain, allodynia, and hyperalgesia; QST was not recommended as a stand-alone test for the diagnosis of neuropathic pain, but it was considered valuable when taken in a clinical context to provide information about the functional status of the somatosensory system.^120^

Overall, QST studies of patients with TMD have found that enhanced pain sensitivity is associated with subsequent development of TMD^121^ and that TMD is associated with various abnormalities in somatosensory profiles compared to controls,^122,123^ although the specific QST parameters found to be abnormal are inconsistent across studies. Although various QST measures have been correlated with subjective pain report, QST is known to be highly variable even among healthy individuals, and even wider interindividual variability has been observed among patients with TMD.^124^ In addition, sensory abnormalities have been observed in patients with TMD at body sites outside the painful facial region.^122,125^ These findings may be reflective of centralized pain phenomena,^126^ which can be present in multiple chronic overlapping pain conditions. From the OPPERA cohort, pressure pain thresholds (PPTs) were found to be weak predictors of TMD onset but were found to be significantly decreased at the time of TMD onset; in addition, PPTs were persistently lower in patients with ongoing TMD symptoms but tended to normalize in cases of symptom resolution.^127^ A more recent study of the OPPERA cohort showed that individuals who transition from control to TMD show the greatest reductions in PPT over a period of 5 to 7 years.^128^ Thus, it appears that the sensitivity and specificity of QST are rather poor for it to be useful as a diagnostic tool in TMD, but it could be more valuable as a monitoring biomarker to track the development or maintenance of sensory abnormalities over time.

In TN, routine neurological evaluation is often normal. QST has identified subtle, subclinical sensory abnormalities in patients with TN using comprehensive QST panels^129–133^ but, as with TMD, the specific abnormalities within those panels vary across studies. Although QST is generally considered more useful in the assessment of neuropathic pain conditions (e.g., TN), somatosensory deficits still occur in nonneuropathic pain conditions (e.g., TMD). Therefore, the diagnostic resolution of QST in distinguishing between neuropathic and nonneuropathic pain is low. As noted above, QST may be more practical as a monitoring biomarker, particularly in the context of assessing somatosensory function before and after treatment. A study of patients with TN before and after decompression surgery found that although pain and masticatory function improved after surgery, QST identified the development of subtle new sensory deficits following surgery.^134^ QST could also serve as a predictive biomarker, forecasting potential efficacy to various treatment options.

## Conclusions

As the demand for precision medicine techniques and personalized pain management grows, the field of pain biomarker research will continue to expand. The spectrum of chronic pain conditions typified by TMD and TN demonstrates the various challenges and exciting opportunities in pain biomarkers. Although there are currently no validated, established biomarkers for pain, promising genetic, molecular, neuroradiological, and psychophysical strategies are currently being explored in both TMD and TN. Most of the potential biomarkers in these conditions are still in the early stages of biomarker discovery and verification. An incomplete, evolving knowledge of the mechanisms of chronic facial pain and imperfect (or, in the case of omics, previously unavailable) measurement techniques have been barriers in the earlier stages of biomarker development. As we have seen in the examples above, the clinical heterogeneity of pain patient populations, small sample sizes, and insufficiently characterized clinical cohorts in both TMD and TN can present challenges to successful biomarker verification and validation. Furthermore, it remains to be seen whether these potential biomarkers can fulfill considerations of reliability and practicality (e.g., cost and speed) to bridge the gap from biomarker validation to implementation. However, as researchers learn more about facial pain pathophysiology, refine assay techniques and technologies, and assemble larger, well-defined clinical cohorts, we may soon have not one, but several, new facial pain biomarkers. The same framework of biomarker development may also be applied and tailored to assess potential biomarkers in other chronic pain states, as well. These tools could provide us with valuable insights into the mechanisms of chronic pain, forecast disease trajectories, predict treatment response, or identify suitable targets for personalized therapies and rational drug design.

Summarizing the evidence for sensory testing, skin biopsy, and functional brain imaging as biomarkers, the Initiative on Methods, Measurement, and Pain Assessment in Clinical Trials noted their potential utility as diagnostic, prognostic, predictive, and pharmacodynamic biomarkers but also called for further standardization and demonstrations of validity and reliability.^135^ Identifying the most useful and successful future biomarkers will require systematic approaches that capitalize on innovative techniques and build on existing knowledge. Despite the trendy appeal of the term “biomarker discovery,” this area of research is not merely a passing trend. Biomarker discovery is a vital goal in pain research, and the results of these efforts will undoubtedly transform the way we approach pain and pain treatment in the future.
